# Supplementary material for: A combinatorial genetic strategy for exploring complex genotype–phenotype associations in cancer
Source: Nat Genet. 2024 Feb 29;56(3):371–6. doi: 10.1038/s41588-024-01674-1 (PMC10937382; doi:10.1038/s41588-024-01674-1)
Supplement: Supplementary file 1 — Supplementary Tables 1–4. [file 41588_2024_1674_MOESM1_ESM.pdf]

nature genetics

Brief Communication

https://doi.org/10.1038/s41588-024-01674-1

A combinatorial genetic strategy for exploring complex genotype–phenotype associations in cancer

In the format provided by the authors and unedited

Supplementary Table 1. Summary of barcoded lentiviral ORF and shRNA constructs.

Barcode (BC) #	*Barcode sequence*	*Encoded gene*	*TRC shRNA clone ID*
1	GCAGATTGTA	*Yap1*	-
2	CAACGTCGAC	ORF control	-
3	TGTTGATTCA	*E2f3*	-
4	TCGCACACAC	*Pparg*	-
5	TAGTTGGCTT	sh*Kdm6a*	TRCN0000331919
6	GGGATCGATC	*Mdm2*	-
7	AGCGTGTACA	*Ccnd1*	-
8	TCGTCCTTGA	sh*Stag2*	TRCN0000295340
9	GAGTCATGTC	sh*Ncor1*	TRCN0000350169
10	GGCCATTCAG	*Pvrl4*	-
11	ATCTACTCGC	*Ywhaz*	-
12	CAAACTACGT	sh*Kmt2d*	TRCN0000239233
13	ACGATATTAG	sh*Rb1*	TRCN0000235830
14	CAAACAATGA	sh*Crebbp*	TRCN0000012725
15	TCGGGACAGA	sh*Ep300*	TRCN0000071207
16	ACCGTTAGAG	*Ccne1*	-
18	ACCCACATGC	sh*Sptan1*	TRCN0000090595
20	CTTTGACTAT	*Zfp703*	-
22	GGTCAAATCG	*Fgfr3* S243C	-
23	CCCAAATGAT	sh*Atm*	TRCN0000360328
24	AGGCCTATCG	*Myc*	-
25	AGACTCGATG	*Erbb2* S311Y	-
26	CTATGCGTCC	sh*Kmt2a*	TRCN0000034426
27	TCGTACGGTT	sh*Kmt2c*	TRCN0000238934
28	GCTGGGAGTA	sh*Pten*	TRCN0000355842
29	GAGTTCTCTA	*Egfr*	-
30	CCTATGAGTT	sh*Cdkn2a*	TRCN0000231227
31	CCAATCTGGG	sh*Arid1a*	TRCN0000071396
32	GAACTGAACT	TRC control	-
33	TGCATCGTTT	*Erbb3* V104L	-
34	GAAATACCTG	*Pik3ca* E545K	-
35	TTTGTTAGGT	sh*Spen*	TRCN0000226288
36	AAACCATACT	sh*Brca2*	TRCN0000349771
37	AACGTGTCGT	sh*Ttn*	TRCN0000362964
38	CGCATAAGCA	sh*Cdk12*	TRCN0000361778
39	GGTCGCAATT	shChd1	TRCN0000096526
40	GGTGGAACTG	sh*Cdkn1b*	TRCN0000294885
41	TGGGTCAGCA	sh*Apc*	TRCN0000244294
43	ATGCTGTAGG	*Prex2*	-
44	TTAAGCGCCT	*Rspo2*	-
45	ATCGGTTCAC	*Foxa1* R261C	-
46	CATGTGGACC	*Spop* F133C	-
47	TGCATGTCCT	*Ctnnb* D32A	-
48	CATTACCAAG	*Erg*	-

49	GGTCACCACA	*Ar*	-
51	ATAGAAACTC	*Etv1*	-
52	AAATGGGTAT	*Braf* G469A	-
54	CATGTCAGTC	sh*Zfhx3*	TRCN0000075411
59	GCGTGGTTCT	*Trp53* R245Q	-

Supplementary Table 2. Efficiency of tumor initiation and tumor latency associated with the organoid transformation assay.

Tissue	*Number of mice inoculated*	*Tumor incidence (%)*	*Average time to 1 cm tumor diameter (months)*
Bladder	20	80%	4.2
Prostate	47	38%	8.9

Supplementary Table 3. Primers used for qPCR studies to quantify relative expression of target genes.

Target gene	*Primer 1 (5' - 3')*	*Primer 2 (5' - 3')*
Kdm6a	ACCTAGTCCTCAGATCATACCA	GCTACTATTAGACAGGCCGTT
Stag2	GGCAGATAAATTTAACCGGCTTC	GCATTATGAAAAGCAGTGATCCTC
Ncor1	ATCCAGCTATGCCCTTTCAC	TGTCTGCCTTGTATTCTCCATT
Kmt2d	TCAGTGCTATCACCCGTACT	CACCTCACACACGATACACTC
Rb1	CCTCAGCCTTCCATACTCAG	CGGAGATATGCTAGACGGTACA
Crebbp	TCACAATCAACATCTCCTTCCC	TGTCGATAGAGTGCTTCTAGAGT
Ep300	CTCAGAAACTGTATGCCACCA	GCATCTCTACCGTCCATCAG
Sptan1	GGAGGTGTATGGTGCGATG	TTGATGGAGTTGAAGGTAGCC
Atm	GTCACAAAGAACCATGCTTGC	CACCTTCGCAACCTCAAGA
Kmt2a	GAAGATGCCTGGAAGTCACT	TGCTCAATCAGAAACACAACG
Kmt2c	AGAAGGATGAAGAGGAAAAGCA	GGTGGTGTAGGAGGAAGAGAG
Pten	CACTGCTGTTTCACAAGATGATG	TTCACCTTTAGCTGGCAGAC
Cdkn2a	GTGCGATATTTGCGTTCCG	CTCTGCTCTTGGGATTGGC
Arid1a	CCTCTATCGCCTCTATGTGTCT	GCACTGCTTGATGTACCCA
Spen	CGACTACTTACCACGACCTTC	CACACACTAGCGATATCACAGT
Brca2	GATGCCTAAACCCAGAAAGAGT	TGTGTCATCCCTCTCCAGTATC
Ttn	CAATGGATCTGGACAAGCGA	CACTCTCACTTGGAGTCTCAC
Cdk12	AACAGACCCTACAGAGTGACT	TCGACGTTTCTTACTCCACAA
Chd1	CGCCCAGCTTCATCTAATAGTG	CATCATTGTGCTTCTTCCTCTTG
Cdkn1b	GAGCAGACGCCCAAGAAG	GCAGTGATGTATCTAATAAACAAGGA
Apc	AGAATGAAGGTCAAGGAGTGG	TACTAGAACTCAAAACACTGGCT
Actb	GATTACTGCTCTGGCTCCTAG	GACTCATCGTACTCCTGCTTG
Zfhx3	AACAACAAGATCCACCTCCAG	ACTAGGCATAACCATCTCAGGA
Ubc	AACATCCAGAAAGAGTCCACC	CATTCTCTATGGTGTCACTGGG
Yap1	CCAGACGACTTCCTCAACAGTG	GCATCTCCTTCCAGTGTGCCAA
E2f3	GTCCAGAAGAGACGGAAACACAC	GCTGTAGAAACCGAGCAGTCAC
Pparg	GTACTGTCGGTTTCAGAAGTGCC	ATCTCCGCCAACAGCTTCTCCT
Mdm2	CCGAGTTTCTCTGTGAAGGAGC	GTCTGCTCTCACTCAGCGATGT
Ccnd1	GCAGAAGGAGATTGTGCCATCC	AGGAAGCGGTCCAGGTAGTTCA
Pvrl4	CAAATGCCTGAGTGAAGGACAGC	TAGACGCCACTGTGCTCAGTAG
Ywhaz	CAGAAGACGGAAGGTGCTGAGA	CTTTCTGGTTGCGAAGCATTGGG
Zfp703	GCTCCCTATCAGGGTCCTGAAG	CCAAAGGACTCTTCTTGGCGTC
Fgfr3	ACAGGTGGTCATGGCAGAAGCT	CTCCATCTCAGATACCAGGTCC
Myc	TCGCTGCTGTCCTCCGAGTCC	GGTTTGCCTCTTCTCCACAGAC
Erbb2	GACCTCAGTGTCTTCCAGAACC	TGCGGTGAATGAGAGCCAATCC
Egfr	GGACTGTGTCTCCTGCCAGAAT	GGCAGACATTCTGGATGGCACT
Erbb3	AGGCTCATTGCTTCTCCTGCCA	GAAAATGGGCGCATCGAGCACA
Pik3ca	CACCTGAACAGACAAGTAGAGGC	GCAAAGCATCCATGAAGTCTGGC
Ccne1	AAGCCCTCTGACCATTGTGTCC	CTAAGCAGCCAACATCCAGGAC
Foxa1	GCCTTACTCCTACATCTCGCTC	CTGCTGGTTCTGGCGGTAATAG
Spop	GCTGTCCAAAGAGTGAAGTTCGG	GCACGAACCTATAAGCTCGCTG
Ctnnb1	GTTCGCCTTCATTATGGACTGCC	ATAGCACCCTGTTCCCGCAAAG
Erg	GGATGTGACCTCCAAACAGGAC	CAGACCCATTGAGTTGGCACAC
Ar	CCTTGGATGGAGAACTACTCCG	TCCGTAGTGACAGCCAGAAGCT

Etv1	TCCTGGCTCATCCAAGCAGAAC	CGGTACATTCCAGGCTCTTGCT
Trp53	TGAAAATGTCTCCTGGCTCAG	CTAGCATTCAGGCCCTCATC

Supplementary Table 4. Primers used for 2° PCR to incorporate dual-indexed Illumina sequencing adaptors for bulk amplicon sequencing.

Name	***Primer sequence (5' - 3')***
i7_7005	CAAGCAGAAGACGGCATACGAGATGTGAATATGTCTCGTGGGCTCGGAGATGTG
i7_7006	CAAGCAGAAGACGGCATACGAGATACAGGCGCGTCTCGTGGGCTCGGAGATGTG
i7_7007	CAAGCAGAAGACGGCATACGAGATCATAGAGTGTCTCGTGGGCTCGGAGATGTG
i7_7008	CAAGCAGAAGACGGCATACGAGATTGCGAGACGTCTCGTGGGCTCGGAGATGTG
i7_7015	CAAGCAGAAGACGGCATACGAGATTCTCTACTGTCTCGTGGGCTCGGAGATGTG
i7_7016	CAAGCAGAAGACGGCATACGAGATCTCTCGTCGTCTCGTGGGCTCGGAGATGTG
i7_7017	CAAGCAGAAGACGGCATACGAGATCCAAGTCTGTCTCGTGGGCTCGGAGATGTG
i7_7018	CAAGCAGAAGACGGCATACGAGATTTGGACTCGTCTCGTGGGCTCGGAGATGTG
i7_7023	CAAGCAGAAGACGGCATACGAGATGCAGAATTGTCTCGTGGGCTCGGAGATGTG
i7_7024	CAAGCAGAAGACGGCATACGAGATAACCGCGGGTCTCGTGGGCTCGGAGATGTG
i7_7025	CAAGCAGAAGACGGCATACGAGATACTAAGATGTCTCGTGGGCTCGGAGATGTG
i7_7026	CAAGCAGAAGACGGCATACGAGATGTCGGAGCGTCTCGTGGGCTCGGAGATGTG
i5_5001	AATGATACGGCGACCACCGAGATCTACACAGCGCTAGTCGTCGGCAGCGTCAGATGTGT
i5_5002	AATGATACGGCGACCACCGAGATCTACACGATATCGATCGTCGGCAGCGTCAGATGTGT
i5_5007	AATGATACGGCGACCACCGAGATCTACACACATAGCGTCGTCGGCAGCGTCAGATGTGT
i5_5008	AATGATACGGCGACCACCGAGATCTACACGTGCGATATCGTCGGCAGCGTCAGATGTGT
i5_5009	AATGATACGGCGACCACCGAGATCTACACCCAACAGATCGTCGGCAGCGTCAGATGTGT
i5_5010	AATGATACGGCGACCACCGAGATCTACACTTGGTGAGTCGTCGGCAGCGTCAGATGTGT
i5_5013	AATGATACGGCGACCACCGAGATCTACACAACCGCGGTCGTCGGCAGCGTCAGATGTGT
i5_5014	AATGATACGGCGACCACCGAGATCTACACGGTTATAATCGTCGGCAGCGTCAGATGTGT
