# Supplementary material for: A combinatorial genetic strategy for exploring complex genotype–phenotype associations in cancer
Source: Nat Genet. 2024 Feb 29;56(3):371–6. doi: 10.1038/s41588-024-01674-1 (PMC10937382; doi:10.1038/s41588-024-01674-1)
Supplement: Supplementary file 2 — Reporting Summary [file 41588_2024_1674_MOESM2_ESM.pdf]

nature portfolio

Corresponding author(s): John K. Lee

Last updated by author(s): Jan 3, 2024

Reporting Summary

Nature Portfolio wishes to improve the reproducibility of the work that we publish. This form provides structure for consistency and transparency in reporting. For further information on Nature Portfolio policies, see our Editorial Policies and the Editorial Policy Checklist.

Statistics

For all statistical analyses, confirm that the following items are present in the figure legend, table legend, main text, or Methods section.

n/a	Confirmed	
☐	☒	The exact sample size (*n*) for each experimental group/condition, given as a discrete number and unit of measurement
☐	☒	A statement on whether measurements were taken from distinct samples or whether the same sample was measured repeatedly
☐	☒	The statistical test(s) used AND whether they are one- or two-sided<br>*Only common tests should be described solely by name; describe more complex techniques in the Methods section.*
☒	☐	A description of all covariates tested
☐	☒	A description of any assumptions or corrections, such as tests of normality and adjustment for multiple comparisons
☐	☒	A full description of the statistical parameters including central tendency (e.g. means) or other basic estimates (e.g. regression coefficient) AND variation (e.g. standard deviation) or associated estimates of uncertainty (e.g. confidence intervals)
☐	☒	For null hypothesis testing, the test statistic (e.g. *F*, *t*, *r*) with confidence intervals, effect sizes, degrees of freedom and *P* value noted<br>*Give P values as exact values whenever suitable.*
☒	☐	For Bayesian analysis, information on the choice of priors and Markov chain Monte Carlo settings
☐	☒	For hierarchical and complex designs, identification of the appropriate level for tests and full reporting of outcomes
☒	☐	Estimates of effect sizes (e.g. Cohen's *d*, Pearson's *r*), indicating how they were calculated

Our web collection on statistics for biologists contains articles on many of the points above.

Software and code

Policy information about availability of computer code

Data collection

The single-cell or bulk DNA amplicon sequencing data collected for this manuscript was generated using a custom panel designed for the Mission Bio Tapestri to amplify segments of ten mouse genes at two exons each, the 5’ and 3’ lentiviral barcodes, and lentiviral GFP. Amplicon DNA libraries generated using Tapestri were sequenced on an Illumina MiSeq or HiSeq 2500 with 150 bp paired-end reads in the Fred Hutchinson Cancer Center Genomics Shared Resource.
The bulk RNA sequencing libraries were prepared using a SMARTer Stranded Total RNA-Seq Kit v3 - Pico Input Mammalian (Takara Bio) and sequenced on an Illumina NovaSeq 6000 using a NovaSeq S4 flow cell with 100 bp paired end reads by MedGenome, Inc.

Data analysis

Data and statistical analysis were performed using Microsoft Excel Office vl6.65 and Graph Pad Prism v9.4.l.
Standard statistical tests were used to analyze biological data including Student's t test,W ilcoxon rank-sum test,F isher's exact test,t wo-way ANOVA with post hoc Tukey's or Sidak's multiple comparisons.
Results for immunohistochemical (IHC) analysis were plotted using QuPath 0.2.3.
Results from flow cytometric analysis were acquired using BD FACSCanto and Sony SH800 Cell Sorter instruments.
Flow data were analyzed using FlowJo 10.8.0.

Bulk RNA-seq and single-cell or bulk DNA amplicon sequencing data were analyzed on a Linux workstation using R v4.l.0.

The sequencing data reported in the manuscript was processed using the following tools or packages: RStudio v4.1.0,biomaRt package v2.24.1,the ConsensusMIBC package v1.1,pheatmap package v1.0.12,prcomp package v3.6.2,factoextra package v1.0.7,ggpubr package v0.6.0,D GEobj.utils package v1.0.6,scale: Scaling and Centering of Matrix-like Objects base package v3.6.2,ggplot2 v3.4.1,DESeq2 package v1.38.3,Gene Set Enrichment Analysis 4.3.1,samtools v1.11,BWA-mem v0.7.17-r1188,Bowtie2 v2.4.4,Cutadapt v4.1,UMI-tools vl.0.0,p ython 3.7,toil-rnaseq v4.1.2,sva (ComBat-seq) v3.36.0)

For functional experiments, each was repeated at least three times independently and results were expressed as mean± SD or mean ± SEM.

For manuscripts utilizing custom algorithms or software that are central to the research but not yet described in published literature, software must be made available to editors and reviewers. We strongly encourage code deposition in a community repository (e.g. GitHub). See the Nature Portfolio guidelines for submitting code & software for further information.

Data

Policy information about availability of data

All manuscripts must include a data availability statement. This statement should provide the following information, where applicable:

- Accession codes, unique identifiers, or web links for publicly available datasets
- A description of any restrictions on data availability
- For clinical datasets or third party data, please ensure that the statement adheres to our policy

Sequencing data pertaining to this study is available from Gene Expression Omnibus (GEO) as SuperSeries GSE229783. RNA-seq data from FHBT models is available from accession number GSE229780. Bulk DNA amplicon sequencing data from lentiviral library representation stud-ies and from FHBT models are available from accession numbers GSE231542 and GSE229781, respectively. Single-cell DNA amplicon sequencing data related to determining the unique proviral copies per cell after lentiviral transduction across a range of MOIs is available from accession number GSE231543. Single-cell DNA amplicon sequencing data from FHBT models and enriched cells from the tumor model with prostate adenocarcinoma and focal pleomorphic giant cell carcinoma is available from accession number GSE229782.

Research involving human participants, their data, or biological material

Policy information about studies with human participants or human data. See also policy information about sex, gender (identity/presentation), and sexual orientation and race, ethnicity and racism.

Reporting on sex and gender	NA
Reporting on race, ethnicity, or other socially relevant groupings	NA
Population characteristics	NA
Recruitment	NA
Ethics oversight	NA

Note that full information on the approval of the study protocol must also be provided in the manuscript.

Field-specific reporting

Please select the one below that is the best fit for your research. If you are not sure, read the appropriate sections before making your selection.

☒ Life sciences ☐ Behavioural & social sciences ☐ Ecological, evolutionary & environmental sciences

For a reference copy of the document with all sections, see nature.com/documents/nr-reporting-summary-flat.pdf

Life sciences study design

All studies must disclose on these points even when the disclosure is negative.

Sample size	In vitro experiments were performed using three independent replicates and each experiment was repeated at least three times. Sample sizes for in vitro experiments were either based on ensuring sufficient statistical power or based on the standard in the field. For in vivo studies, transduced mouse bladder or prostate cells were subcutaneously injected in 5-6 mice and those which formed tumors were collected and screened by histology. Sample size was based on prior experience with dissociated-cell tissue recombination/transplantation assays and inherent variability due to technical complexity and pilot studies of the frequency of transformation of prostate (30-40%) and bladder (70-80%) epithelial cell grafts using the methodology (see Extended Data Table 1). 5-6 grafts from each transformation study ensured the generation of tumors from at least one graft for each experiment.
Data exclusions	No data was excluded from the analyses.
Replication	Experiments have been repeated multiple times using different independent biological samples with similar experimental conditions or otherwise mentioned in the figure legends, main text or methods.
Randomization	Randomization was not applicable as there was no pre-specified comparison of interventions.
Blinding	Blinding was not applied to the study as a therapeutic intervention was not investigated.

Reporting for specific materials, systems and methods

We require information from authors about some types of materials, experimental systems and methods used in many studies. Here, indicate whether each material, system or method listed is relevant to your study. If you are not sure if a list item applies to your research, read the appropriate section before selecting a response.

Materials & experimental systems

n/a	Involved in the study
☐	☒ Antibodies
☐	☒ Eukaryotic cell lines
☒	☐ Palaeontology and archaeology
☐	☒ Animals and other organisms
☒	☐ Clinical data
☒	☐ Dual use research of concern
☒	☐ Plants

Methods

n/a	Involved in the study
☒	☐ ChIP-seq
☐	☒ Flow cytometry
☒	☐ MRI-based neuroimaging

Antibodies

Antibodies used

Antibodies used in this study for FACS:
Human/mouse/bovine integrin alpha 6/CD49f PE-conjugated antibody (FAB13501P, R&D Systems, 1:40);
PE/Cyanine 7 anti-mouse CD325 (Ep-CAM) antibody (118216, Biolegend, 1:40);
CD31 (PECAM-1) monoclonal antibody (390), FITC (11-0311-82,eBioscience, 1:100);
CD45 monoclonal antibody (30-Fll), FITC (11-0451-85, eBioscience, 1:100);
TER-119 monoclonal antibody (TER-119), FITC (11-5921-82, eBioscience, 1:100).

Antibodies used for immunohistochemistry:
Rabbit polyclonal panCK (ab9377, Abcam, 1:100);
Rabbit monoclonal GFP antibody (clone D5.1, Cell Signaling, 1:100);
Rabbit polyclonal p63 antibody (12143-1-AP, Proteintech, 1:200);
Mouse monoclonal p53 antibody (clone 1C12, Cell Signaling, 1:500);
Rabbit monoclonal HOXB13 antibody (clone D7N8O, Cell Signaling, 1:50);
Rabbit polyclonal AR antibody (06-680, Millipore, 1:2,000);
Rabbit monoclonal GATA3 antibody (clone D13C9, Cell Signaling, 1:200);
Rabbit monoclonal CD44 antibody (clone E7K2Y, Cell Signaling, 1:100).

Validation

For each antibody, the validation statement has been taken from the manufacturer's website or data sheet and detailed as follows:

Human/mouse/bovine integrin alpha 6/CD49f PE-conjugated antibody (FAB13501P, R&D Systems) - the antibody is validated to detect human, mouse, and bovine Integrin alpha 6/CD49f. Recognizes an epitope in the extracellular domain of the Integrin alpha 6 subunit.

PE/Cyanine 7 anti-mouse CD325 (Ep-CAM) antibody (clone- G8.8, 118216, BioLegend)- validated to be used by flow and is cited in multiple publication can be seen on this website: https://www.biolegend.com/fr-lu/products/pe-cyanine7-anti-mouse-cd326-ep-cam-antibody-5303.

CD31 (PECAM-1) monoclonal antibody (390), FITC (11-0311-82,eBioscience) - validated by staining in more than 40 publications found on this website: https://www.thermofisher.com/antibody/product/CD31-PECAM-1-Antibody-clone-390-Monoclonal/11-0311-82.

CD45 monoclonal anitbody (30-F11), FITC (11-0451-85, eBioscience) - the 30-F11 antibody has been tested by flow cytometric analysis of mouse bone marrow cells. https://www.thermofisher.com/antibody/product/CD45-Antibody-clone-30-F11-Monoclonal/11-0451-82

TER-119 monoclonal antibody (TER-119), FITC (11-5921-82, eBioscience) - has been tested by flow cytometric analysis of mouse bone marrow cells. https://www.thermofisher.com/antibody/product/TER-119-Antibody-clone-TER-119-Monoclonal/17-5921-82

Rabbit polyclonal panCK (ab9377, Abcam, 1:100) - suitable for: IHC-P, ICC, ICC/IF, Flow Cyt, WB, IHC-Fr. https://www.abcam.com/products/primary-antibodies/wide-spectrum-cytokeratin-antibody-ab9377.html

Rabbit monoclonal GFP antibody (clone D5.1, Cell Signaling, 1:100) - validated for WB and IHC. https://www.cellsignal.com/products/primary-antibodies/gfp-d5-1-rabbit-mab/2956

Rabbit polyclonal p63 antibody (12143-1-AP, Proteintech, 1:200) - validated for WB, IHC, IP and IF. https://www.ptglab.com/products/TP63-Antibody-12143-1-AP.htm

Mouse monoclonal p53 antibody (clone 1C12, Cell Signaling, 1:500) - validated for WB, IHC, flow, and ChIP. https://www.cellsignal.com/products/primary-antibodies/p53-1c12-mouse-mab/2524

Rabbit monoclonal HOXB13 anti-body (clone D7N8O, Cell Signaling, 1:50) - Validated for WB, IP, and IHC. https://www.cellsignal.com/products/primary-antibodies/hoxb13-d7n8o-rabbit-mab/90944

Rabbit polyclonal AR antibody (06-680, Millipore, 1:2,000) - validated for WB and IHC. https://www.emdmillipore.com/US/en/product/Anti-Androgen-Receptor-Antibody,MM_NF-06-680

Rabbit monoclonal GATA3 antibody (clone D13C9, Cell Signaling, 1:200) - validated for WB, IHC, ChIP, IF and flow. https://www.cellsignal.com/products/primary-antibodies/gata-3-d13c9-xp-rabbit-mab/5852

Rabbit monoclonal CD44 antibody (clone E7K2Y, Cell Signaling, 1:100) - validated for WB and IHC. https://www.cellsignal.com/products/primary-antibodies/cd44-e7k2y-xp-rabbit-mab/37259

Eukaryotic cell lines

Policy information about cell lines and Sex and Gender in Research

Cell line source(s)	HEK293T (CRL-3216) were obtained from the American Type Culture Collection and were cultured in DMEM medium supplemented with 10% FBS, 100 U/mL penicillin and 100 µg/mL streptomycin, and 4 mmol/L GlutaMAX (Thermo Fisher).
Authentication	Cell line authentication was done via short tandem repeat (STR) profiling at the IDEXX BioAnalytics, 4011 Discovery Drive, Columbia, MO 65201.
Mycoplasma contamination	All cell lines routinely tested negative for Mycoplasma contamination.
Commonly misidentified lines (See ICLAC register)	No misidentified cell lines were used in this study.

Animals and other research organisms

Policy information about studies involving animals; ARRIVE guidelines recommended for reporting animal research, and Sex and Gender in Research

Laboratory animals	For studies using immunocompromised mice, six- to eight-week-old male NSG (NOD-SCID-IL2Rγ-null) mice were obtained from The Jackson Laboratory and were 2-4 months old when used for the studies.<br>Eight- to twelve-week-old male wild-type C57Bl/6 (C57Bl/6J) mice were obtained from The Jackson Laboratory and were 2-4 months old when used for the studies.
Wild animals	The study did not involve wild animals.
Reporting on sex	Male mice were used for all the experiments.
Field-collected samples	The study did not include field-collected samples.
Ethics oversight	All animal care and studies were performed in accordance with an approved Fred Hutchinson Cancer Center Institutional Animal Care and Use Committee protocol (PROTO000051048) and Comparative Medicine regulations.

Note that full information on the approval of the study protocol must also be provided in the manuscript.

Flow Cytometry

Plots

Confirm that:

☒ The axis labels state the marker and fluorochrome used (e.g. CD4-FITC).

☒ The axis scales are clearly visible. Include numbers along axes only for bottom left plot of group (a 'group' is an analysis of identical markers).

☒ All plots are contour plots with outliers or pseudocolor plots.

☒ A numerical value for number of cells or percentage (with statistics) is provided.

Methodology

Sample preparation	Bladder and prostates from eight- to twelve-week-old male C57BL/6 mice were dissociated into single cells and were stained with antibodies for fluorescence-activated cell sorting on a Sony SH800 Cell Sorter. Bladder urothelial and prostate epithelial cells were sorted and collected based on a Lin(-) CD49f(high) EpCAM(high) immunophenotype. For prostate polymorphic giant cells were analyzed based on forward and side scatter and further staining for nuclear DNA content with Hoechst 33342 dye.
Instrument	Flow cytometric analysis or sorting were performed using BD FACSCanto and Sony SH800 Cell Sorter instruments.
Software	FlowJo 10.8.0 software.

Cell population abundance	Bladder and prostates from eight- to twelve-week-old male C57BL/6 mice were dissociated into single cells and were stained with antibodies for fluorescence-activated cell sorting on a Sony SH800 Cell Sorter. Bladder urothelial and prostate epithelial cells were sorted and collected based on a Lin(-) CD49f(high) EpCAM(high) immunophenotype. For prostate polymorphic giant cells were analyzed based on forward and side scatter and further staining for nuclear DNA content with Hoechst 33342 dye. Cell population abundance is shown in associated flow plots showing the gating strategy.
Gating strategy	Stained prostate and bladder epithelial cells were plotted based on side scatter-area (SSC-A) and FITC (lineage markers) and the lineage-negative population was selected for gating by CD49f-PE and EpCAM-APC. The CD49f(high) EpCAM(high) population was sorted for experimental use.<br><br>Dissociated cells from mixed prostate adenocarcinoma an pleomorphic giant cell carcinoma tumors were plotted based on forward scatter-height (FSC-H) and forward scatter-area (FSC-A) to select single cells. Single cells were then gated based on high SSC-A and high FSC-A to isolate larger cells. After passage in organoid culture, dissociated and stained tumor cells were plotted based on SSC-A and Hoechst 33342 staining. Tumor cells were sorted based on high and low Hoechst 33342 staining.<br><br>The gating strategies for isolating prostate and bladder epithelial cells and the polymorphic giant cells isolation are provided in the Extended Data Figs. 1a, and 8c, respectively.

☒ Tick this box to confirm that a figure exemplifying the gating strategy is provided in the Supplementary Information.
